# Gastric Tropism: Localized Gastric Amyloidosis Presenting as Gastrointestinal Bleeding

**DOI:** 10.14309/crj.0000000000001652

**Published:** 2025-03-28

**Authors:** Nader Mekheal, Lefika Bathobakae, Alisa Farokhian, Yana Cavanagh

**Affiliations:** 1 Internal Medicine, St. Joseph's University Medical Center, Paterson, NJ; 2 Gastroenterology & Hepatology, St. Joseph's University Medical Center, Paterson, NJ; 3 Advanced & Surgical Endoscopy, St. Joseph's University Medical Center, Paterson, NJ

## CASE REPORT

A 78-year-old woman with a history of hypertension, chronic constipation, and diverticulosis presented to the emergency department complaining of coffee-ground emesis and dark stools for 1 day. In the emergency department, the patient was tachycardic but hemodynamically stable. Triage laboratory results revealed hemoglobin of 7.6 g/dL, which improved to 9.4 g/dL after 2 units of packed red blood cells. A computed tomography angiogram of the abdomen and pelvis showed an area of contrast pooling in the jejunum, concerning for active bleeding. The radiologist concluded that it was an artifact from the inferior vena cava filter.

**Figure 1.**
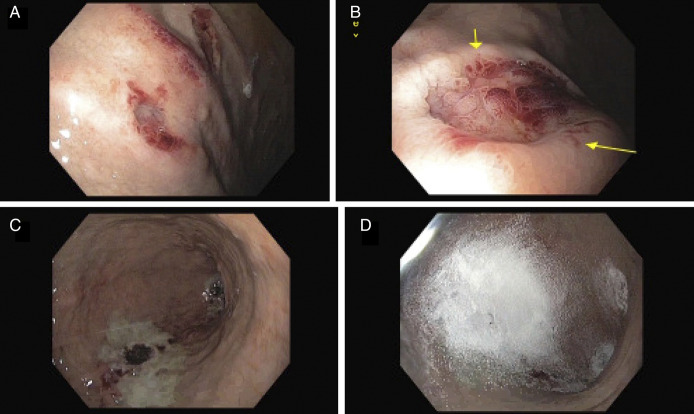
Endoscopic images showing a crated gastric lesion (yellow arrows) with bluish discoloration and stigmata of recent bleeding (A and B). Hemostasis was achieved using epinephrine sclerotherapy, hemospray, and argon plasma coagulation (C and D).

**Figure 2.**
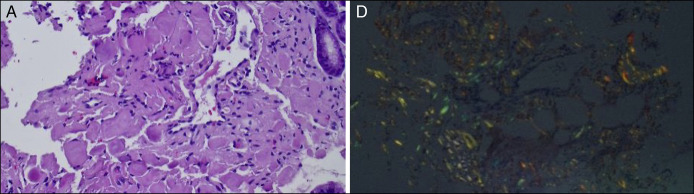
Hematoxylin & eosin staining demonstrating deposition of amyloid fibrous connective tissues in the interstitium with inflammatory cell infiltration (A). Congo red staining showing positivity for amyloid protein (D).

Esophagogastroduodenoscopy showed diffuse bluish discoloration in the gastric fundus/greater curvature with overlying crated lesions (Figure 1). Gastric body lesion measured 15 mm in diameter and actively oozing blood. Hemostasis was achieved using epinephrine injection, hemospray, and argon plasma coagulation. Histopathology of gastric specimens confirmed the presence of the amyloid deposits (Figure 2).

Follow-up tests, including renal ultrasound, echocardiogram, radiography, and urine studies, were unremarkable. The patient was discharged on pantoprazole, with outpatient follow-up for repeat esophagogastroduodenoscopy and possible endoscopic ultrasound. Localized gastric amyloidosis is a rare condition characterized by the deposition of amyloid proteins in the stomach, without systemic involvement.^1,2^ It is an exceptionally rare cause of upper gastrointestinal bleeding, with few cases documented in the medical literature.^2^

## DISCLOSURES

Author contributions: N. Mekheal conceptualized the idea of this image article. L. Bathobakae and A. Farokhian assisted with data curation and writing of the manuscript. Y. Cavanagh edited, fact-checked, and proofread the final version of this case report. L. Bathobakae is the article guarantor.

Financial disclosure: None to report.

Informed consent was obtained for this case report.
